# Development and feasibility testing of a conversational chatbot supporting genetic education and testing for hereditary cancer

**DOI:** 10.1007/s12687-026-00873-z

**Published:** 2026-06-22

**Authors:** Samuel Tundealao, Emily Heidt, Sherry Grumet, Marc D. Schwartz, Beth N. Peshkin, Jinghua An, Scott T. Walters, Lindsay O’Boyle, Deborah Toppmeyer, Anita Y. Kinney

**Affiliations:** 1 Rutgers Cancer Institute, 195 Little Albany St, New Brunswick, NJ 08901 USA; 2 School of Public Health, Rutgers University, 683 Hoes Lane West, Piscataway, NJ 08854 USA; 3 Lombardi Comprehensive Cancer Center, Georgetown University, 3800 Reservoir Rd NW, Washington, DC 20007 USA; 4 Jess and Mildred Fisher Center for Hereditary Cancer and Clinical Genomics Research, 2115 Wisconsin Ave NW, Washington, DC 20007 USA; 5 Indiana University School of Nursing, 600 Barnhill Dr, Indianapolis, IN 46202 USA; 6 University of North Texas Health Science Center, 3500 Camp Bowie Blvd, Fort Worth, TX 76107 USA

**Keywords:** Cancer, Genetic testing, Genetic education, Digital guides, Chatbots

## Abstract

This study describes the development and feasibility testing of a digital health guide (DHG) to streamline genetic education, reduce barriers, and promote informed genetic testing (GT) decisions among cancer survivors. This study reports on the DHG’s development, usability testing, acceptability, feasibility, and preliminary efficacy in improving genetic counseling (GC) and GT access for cancer survivors. Guided by the Ottawa Decision Support Framework, the DHG prototype was developed following community engagement with cancer patients and at-risk relatives from diverse sociodemographically backgrounds. It was refined through user (content-focused) and usability (functionality-focused) testing. Pilot trial participants provided data through semi-structured interviews and usability assessments. Qualitative data were analyzed using the Framework Method. The preliminary impact of the DHG on GC and GT uptake, and informed decision-making, was assessed in a feasibility and accessibility trial. The Chatbot Usability Questionnaire score for the DHG was 70.3 (IQR = 12.5), indicating good acceptability. The DHG also facilitated GT uptake (73.3%) compared to enhanced usual care (EUC; 7.7%). Pretest GC was requested by 1 of 13 patients in the EUC arm, while no request (0 of 15 patients) was made in the DHG arm. Users’ feedback led to clearer language, improved navigation, and stronger messaging regarding data security. DHG participants had lower decisional conflict (33.37 ± 21.09) and decision regret (17.5 ± 16.50) than those in the EUC arm (53.25 ± 22.66 and 37.08 ± 17.38, respectively). The digital intervention is feasible, acceptable, and a promising strategy for expanding GT access and promoting informed decision-making. Further testing in a definitive randomized controlled trial is warranted. Clinical trial registration. This study was preregistered at the NIH clinical trial registry (https://clinicaltrials.gov/study/NCT06184867).

**Supplementary Information:**

The online version contains supplementary material available at https://doi.org/10.1007/s12687-026-00873-z.

## Introduction

Research discoveries, technological innovations, and the decreasing cost of genetic testing (GT) have made it feasible to integrate clinical genomics into cancer treatment and prevention. National guidelines now recommend universal germline GT for all women diagnosed with epithelial ovarian cancer, all men with high-risk, regional, or metastatic prostate cancer, all patients with pancreatic cancer, and colorectal, breast, and endometrial cancer patients meeting specific risk criteria (NCCN 2025a, 2025b).

These expanded guidelines reflect the fact that germline pathogenic variants (PV) are common in these cancers, ranging from 15 to 22% and even up to 30% in some settings (Brianese et al. 2018; Dal Buono et al. 2023; Gonzalez-Angulo et al. 2011; Norquist et al. 2016; Uson et al. 2022). The identification of a PV can inform treatment for newly diagnosed cancers, progression, and recurrence, as well as surgical decisions to reduce the high risk of secondary cancers (Howard-Mcnatt 2021; Kwon 2024; Renkonen-Sinisalo et al. 2017; Teppala et al. 2024). It can help facilitate cascade testing and guide both primary and secondary cancer prevention in at-risk relatives (Offit et al. 2020; Schmidlen et al. 2022b). Despite clear national guidelines and documented health benefits, GT utilization among high-risk cancer survivors is suboptimal, with fewer than 50% referred for GT (Kurian et al. 2021; Muller et al. 2018). Less than 10% of individuals with PV in the United States have been tested, leaving millions of cancer survivors and their at-risk relatives unaware of their increased risk (Childers et al. 2017; Evans and Manchanda 2020).

Research has documented a variety of individual-, familial-, clinician-, system-, and societal-level barriers to testing (Cragun et al. 2017; Levine et al. 2024). Key barriers to testing include low patient awareness, cost concerns, lack of clinician knowledge about whom to test and the process of testing, low rates of clinician referral, a shortage and lack of timely access to cancer genetic counselors, multiple steps needed to access GT, and suboptimal family communication that subsequently limits cascade testing of at-risk relatives (Khan et al. 2021). The traditional clinical pathway for GT requires a visit with a trained health professional for pretest genetic counseling (GC) and another potential follow-up visit for positive test result. Evidence suggests that this approach (of pretest GC) is a major barrier and does not meet the needs of many survivors (Joseph et al. 2017, 2019; Kinney et al. 2023). Thus, there is an urgent need to develop and test new and streamlined models of genome-based care that are more responsive to patients’ needs, improve awareness and access, and do not overburden scarce GC resources.

Given these barriers, integrating digital technology such as chatbots to proactively deliver pretest genetic education, facilitate GT, and disclose test results is an innovative strategy to reduce barriers, extend reach, enhance access, and foster informed decision-making. Automated approaches have the potential to deliver high-quality pretest genetic education while reserving resource-intensive GC for results disclosure and interpretation of positive results, where it is most likely to impact care (Schneider et al. 2025). Web-based pretest genetic education has been shown to be noninferior to conventional GC for psychological distress (Biesecker et al. 2018), resulting in higher GT testing rates in probands (Swisher et al. 2023) and higher rates of cascade testing (Grisham et al. 2024). Digital interventions such as chatbots, conversational agents, and intelligent virtual assistants use human-created decision trees and artificial intelligence (AI) technology to simulate human conversations using text, audio, and graphics (Garg et al. 2018). Digital interventions have been effectively used for health communication in adults of all ages, races, and ethnic backgrounds. They have successfully improved the understanding of cancer susceptibility testing (Wolfe et al. 2015), facilitated informed consent for GT, and provided shareable information after result disclosure (Schmidlen et al. 2019).

However, to date, the efficacy of chatbots for delivering pretest genetic education for cancer survivors and GT results disclosure has not been rigorously evaluated (Schmidlen et al. 2019). Previous and ongoing clinical studies have evaluated chatbots for tailored cancer risk assessment and/or GC and GT outcomes among individuals without a history of cancer, biobank participants, narrowly defined subgroups of cancer survivors (e.g., ages 18–49, specific racial or ethnic groups, a single cancer type, a single syndrome, or one sex), offer exclusively pretest GC, result disclosure among biobank participants, or population‑level screening (Coen et al. 2025; Kaphingst et al. 2024; Nazareth et al. 2021; Welch et al. 2020). However, our study’s inclusion of a broader clinical and demographic population of cancer survivors who received electronic health record case ascertainment and proactive outreach with a digital health guide, DHG (i.e., chatbot) that facilitates GT and provides both pre‑ and post‑test GC is novel.

The **C**hoices **A**bout genetic **T**esting **A**nd **L**earning **Y**our risk with **S**mart **T**echnology (CATALYST) study, therefore, aimed to (1) incorporate feedback from focus groups of socio-demographically diverse cancer patients and their relatives in the design of a DHG prototype; (2) finalize and optimize the development of the theoretically and community informed DHG (an AI-enabled chatbot) through two phases of testing: user (content-focused) and usability (functionality-focused); and (3) assess the feasibility and acceptability of the DHG intervention vs. enhanced usual care (EUC) among cancer survivors through a randomized pilot trial.

## Materials and methods

The study was approved by the Rutgers University Institutional Review Board (#Pro2023000964). All participants gave informed consent before participation. This study follows the CONSORT extension for reporting randomized pilot studies (Eldridge et al. 2016).

### Development of the prototype DHG

#### Formative research

We conducted focus groups to explore barriers and facilitators of GT uptake and elicit recommendations for the development of a chatbot to support cancer genetic education and testing. Research study staff identified patients who visited the Rutgers Cancer Institute and had been diagnosed with cancer, a PV or were family members of cancer patients. Cancer patients also referred their family members to participate, and family members were also recruited through the community and organizations that provide support for family members of cancer patients.

We conducted 8 focus groups with a total of 51 participants, comprising 3 focus groups of individuals with PV (*BRCA1/2* and Lynch syndrome, *n* = 24), 3 groups of Black (*n* = 13) and Hispanic (*n* = 5) cancer patients, and 2 groups of relatives of Black (*n* = 3) and Hispanic (*n* = 6) cancer patients (see eTable 1 Supplement for the focus group participants sociodemographic and clinical characteristics). The primary themes discussed by the participants encompassed familial involvement in GT from both patient and relative perspectives, barriers and facilitators of GT decision-making, the mitigation of mistrust and stigma around GT, barriers (such as extra clinic appointments with genetic counselors, geographic distance) to GT uptake, and facilitators (such as perceived personal and family health benefits, personalized medical care based on results) of GT uptake.

Participants enthusiastically supported the development of a chatbot to support hereditary cancer genetic education and testing, and they provided specific recommendations for content. The key intervention features suggested included genetic literacy, measures to enhance trust and credibility, patient testimonials, understandable risk information, actionable steps for GT, and an easy user interface. The participants preferred a Rutgers-affiliated portal because of privacy concerns and cited it as trustworthy. The suggested chatbot functionalities include an accessible interface, an interculturally competent avatar, and options for text and voice communication, as well as referrals for GC, testing, and support groups. The Black and Hispanic groups proposed acknowledging the generational gap in families regarding technology, providing users with choices for receiving GT results (e.g., mail to home address, send to email address, or call with a genetic counselor), and employing social marketing strategies, such as testimonials and measures to instill trust (e.g., videos of patients with negative/positive GT result or their relatives). They also emphasized the need for multi-media elements (text, voice graphics, videos) and socially relevant content.

#### Theoretical framework

The Ottawa Decision Support Framework (ODSF) underpins our intervention and measurement approach (Hoefel et al. 2020a, b; Stacey et al. 2020). The ODSF provides a structure to facilitate and evaluate informed decision-making by ensuring that decisions are based on relevant knowledge and attitude alignment. Congruent with the ODSF, proactive genetic education via the DHG covers key components of typical GC, with the intent of facilitating awareness, knowledge, attitude clarification, alignment, and informed GT decisions, resulting in increased GT uptake and improved decision-making outcomes. In contrast, the lack of proactive genetic education and low uptake of GC in the EUC arm will likely yield lower GT uptake and poorer decision-making outcomes compared to the DHG arm.

#### Initial prototype development

**Fig. 1:**
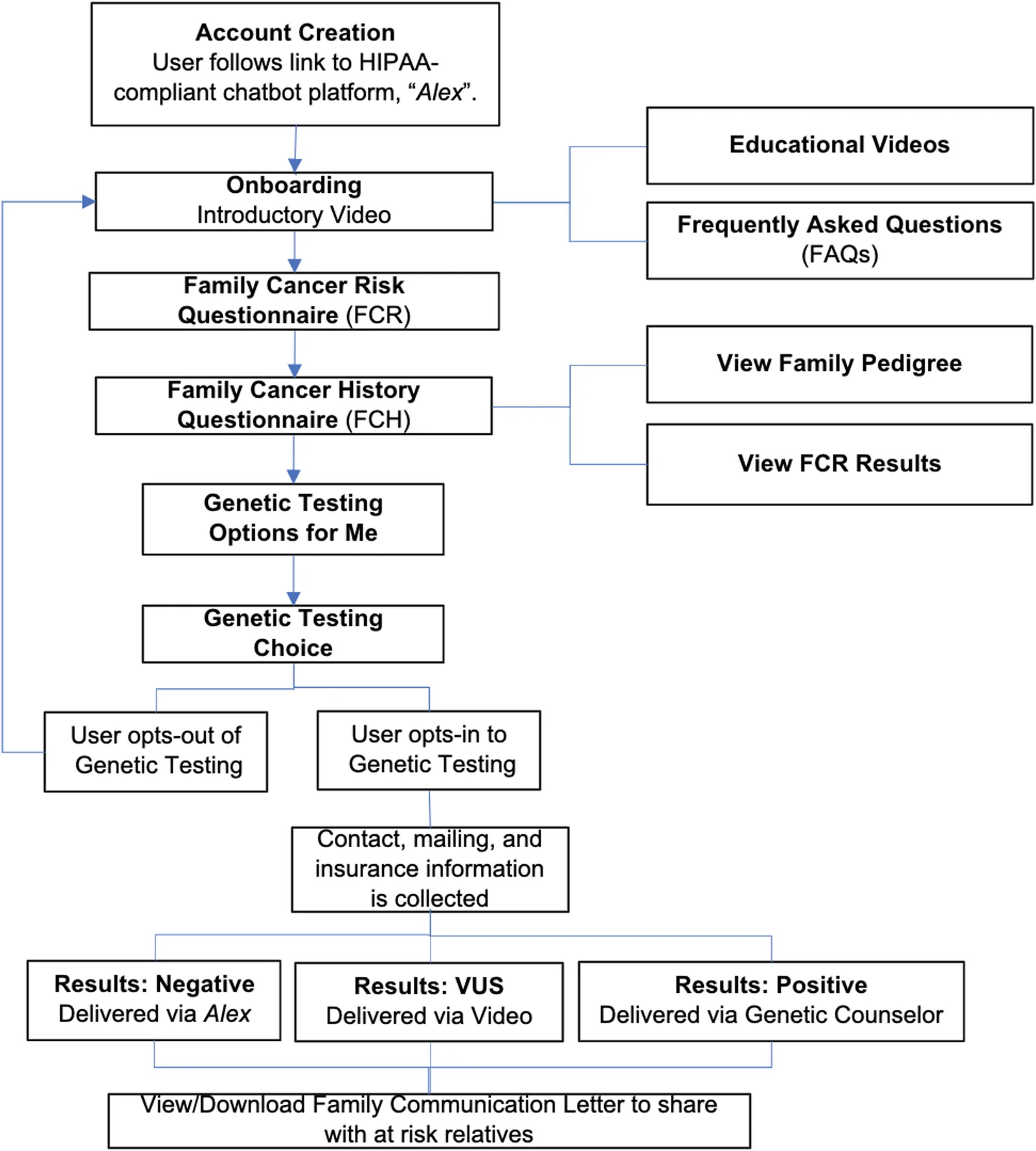
Digital Health Guide user journey before the user and usability testing

The DHG, “Alex”, was designed as a structured, HIPAA-compliant guided user journey (Fig. 1) with required and optional modules. The required modules include log-in, account creation, onboarding, introduction to hereditary cancer, GT readiness, GT choice, GT result delivery, and GT family communication letter. The optional modules include educational and patient testimonial videos, frequently asked questions (FAQ), and cancer family history questionnaire. The cancer family history questionnaire was made optional because we prioritized NCCN cancer-based eligibility, which is reliably captured in the medical record, aligns with national guidelines, and has been shown to outperform family history in identifying patients with actionable hereditary cancer risk. In addition, completing a detailed pedigree early created additional participant burden, and many users lacked complete family information and preferred to decide about testing first. Additional DHG platform features include user preferences, voice chat, tabs (chat vs. questionnaire), menu option, and knowledge base. Participants spent 10.5 min (SD = 5.7) completing the pre-GT required modules and 12 min (SD = 9) on the post-GT required modules, while the optional modules varied in duration depending on user preference and level of engagement. For example, the optional educational video library includes seven videos with 4 patient testimonials, each approximately 2–4 min in length, while the optional cancer family history questionnaire could range from 10 min to 1 h, depending on the complexity and completeness of the participant’s family cancer history. Alex utilizes a Retrieval-Augmented Generation framework powered by the GPT-4 Large Language Model. The system is tailored and guard-railed for Rutgers’ needs by limiting reliance on the model’s general knowledge and instead leveraging structured, credible information sourced from national resources and vetted by the study team’s clinical experts. Users’ data are protected through multiple layers of security built into the DHG. The HIPAA-compliant DHG requires secure password-protected accounts and is hosted within Rutgers-affiliated medical infrastructure. Additionally, because of the restricted and vetted knowledge base, the DHG minimizes inappropriate data handling and prevents inaccurate responses.

Alex’s functionalities are organized into two main tabs: Chat and Questionnaires. The Chat tab stores user interactions, allowing participants to review their conversation history, while the Questionnaires tab contains various assessments that become available as users progress. This section enables users to track their progress, complete questionnaires, and download personalized documents like risk summaries and family trees. There is a toggle option for participants to switch between enabling and disabling the voice chat option. A permanent Menu offers an overview of key checkpoints, aiding navigation. To enhance user experience, Alex features interactive tools like educational resources and personalized content. Participants can access videos, FAQs, and questionnaires at any point and download communication aids, such as “Questions for their Doctor” and a “Family Communication Letter,” to facilitate discussions about hereditary cancer and their GT results with healthcare providers and relatives, respectively.

Alex also facilitates GT and relays GT results. When a participant indicates that they would like GT, the DHG offers two options: proceed directly to GT or schedule a GC session with a certified genetic counselor (by phone, televideo, or in-person). The DHG notifies study staff when a patient requests GT, and a testing kit is mailed to them from a commercial lab, including instructions, consent forms, and prepaid return packaging. GC staff assist with obtaining insurance authorization or financial aid through medical assistance programs if needed. Since the study participants met the NCCN testing criteria and the lab was in-network with most health plans, all the DHG participants used their insurance coverage, and none had any out-of-pocket costs. None of the participants elected the patient‑pay (self‑pay) option of $250. The DHG alerts patients via email and/or text (depending on patient preference) when their GT results are available. GT results are disclosed by a certified genetic counselor for patients who have a PV or by mail/email for negative or VUS results. GT results are also available via the patient portal in the electronic health record (EHR) and the DHG platform. Participants received their results, a clinical summary letter, and a family sharing letter via the DHG platform as well as through postal mail and email, and were encouraged to follow up with their physician or schedule additional GC sessions if desired.

### User and usability testing

The initial prototype was refined through user and usability testing. User testing sessions addressed content and design, such as clarity, formatting, and engagement, while usability testing focused on navigation, error management, and the time it took to complete a task.

#### Participant selection

Participants were approached through multiple recruitment pathways, including identification via the electronic health record with oncologist permission-to-contact procedures, clinician referral at Rutgers Cancer Institute sites, flyers posted in clinic settings, and outreach through online and community cancer support groups. Eligible individuals were then invited directly by email, phone, or in person to complete the screening and schedule the HIPAA-compliant Zoom or Microsoft Teams interview (eFigure 1, Supplement).

Eligibility criteria included (1) age ≥18 years, (2) ability to read and speak English, and (3) a personal history of either ovarian, breast, pancreatic, colorectal, endometrial, or prostate cancer. The recommended sample size for user and usability testing is 3–5 participants, with additional recruitment until thematic saturation is reached (Guest et al. 2006; Pernice and Nielson 2012). Saturation is reached when no new or significant information is gathered from additional data collection. Saturation was achieved for this study after 14 interviews (6 user and 8 usability).

#### Procedures

We employed a mixed-methods approach, combining flexible, open-ended exploration of participants’ experiences with structured, quantifiable usability testing data (eTable 2, Supplement). In-depth, semi-structured interviews utilizing think-aloud techniques captured participants’ preferences and expectations and identified platform issues. Interviews for both user and usability testing were conducted remotely using HIPAA-compliant Zoom or Microsoft Teams. Each interview was conducted individually, with one research coordinator interviewing one participant at a time. Interviews were typically scheduled for 60 to 90 min. Participants were provided with $75 gift card for participation.

#### Measures

The user and usability testing interview guides incorporated both open-ended and structured questions, covering participants’ health and medical history, device usage, and sociodemographic information. Quantitative measures included the Chatbot Usability Questionnaire (CUQ), which was used to evaluate Alex’s acceptability (Holmes et al. 2019). The CUQ is a validated, standardized instrument designed to assess users’ perceived usability of conversational agents. The CUQ consists of 16 items adapted from established usability frameworks, including the System Usability Scale, and evaluates multiple domains such as ease of use, clarity of responses, efficiency, engagement, and overall satisfaction with the chatbot interaction. Items are rated on a Likert scale, with higher scores indicating greater perceived usability. The CUQ has demonstrated good internal consistency (Cronbach’s alpha = 0.90) and construct validity across diverse chatbot applications and is commonly used to evaluate user experience and usability in health-related conversational systems (Holmes et al. 2019, 2023). CUQ has a benchmark score of 68 out of 100 (Boyd et al. 2022; Larbi et al. 2022). The usability testing was conducted as a facilitated, online screenshare session, during which the facilitator directly observed participants as they navigated the platform using a think-aloud approach. Task performance, navigation challenges, and any required assistance were documented in real time. All sessions were audio- and video-recorded to allow for later verification and additional detail during analysis. Participants then completed the CUQ with the facilitator present during the same guided session. Participants were encouraged to navigate the platform independently while thinking aloud. Coordinators offered support only when explicitly requested or when significant issues arose.

#### Data analysis

Interviews were recorded, transcribed, and analyzed using Atlas.ti 24 software. We employed the Framework Method for qualitative data analysis (Gale et al. 2013), combining inductive and deductive approaches to develop themes from participants’ experiences and existing literature. Two coders independently analyzed the transcripts to ensure reliability, followed by batch meetings to review them line by line and resolve discrepancies through consensus. When consensus was not achieved, a third coder adjudicated. Inter-rater reliability was high, with an overall percent agreement of 70.2% and Cohen’s κ = 0.96, indicating almost perfect agreement. Descriptive statistics were used to summarize quantitative sociodemographic and clinical characteristics, along with average CUQ values.

### Pilot-testing of the DHG

The feasibility, acceptability, and preliminary efficacy of the DHG intervention vs. EUC were assessed among cancer survivors through a randomized pilot trial.

#### Participant selection

We used a proactive outreach and clinical informatics approach for recruitment. Participants were identified through the EHR. Eligibility criteria were (1) age ≥18 years, (2) the ability to read and speak English, and (3) a personal history of ovarian, breast, pancreatic, colorectal, endometrial, or prostate cancer that met national criteria for germline genetic testing (NCCN 2025a, 2025b) (eFigure 2, Supplement). Patients were excluded if they lacked internet access, had previous cancer GT, self-reported inability to use the internet independently, or had previously participated in user/usability testing. Since the data are descriptive in nature and will help inform a future definitive randomized trial, a sample size of 30 patients is sufficient for this study (Eldridge et al. 2016; Lewis et al. 2021). It would provide a margin of error of 0.167 when the true proportion is 0.3, a margin of error of 0.178 when the true proportion is 0.4, and a margin of error of 0.182 when the true proportion is 0.5.

#### Procedures

Eligible patients were sent a study information letter (via email, postal mail, and/or patient portal), a study flyer, and an opt-out postcard/toll-free telephone number/email address. Interested patients completed the internet eligibility survey, consent and HIPAA authorization form, and baseline survey. Following completion of the baseline survey, participants were randomized 1:1 to either the EUC or DHG arm. Randomization was implemented in REDCap using a computer-generated algorithm stratified by sex and cancer type, with block randomization (block size = 4) to maintain balance across strata. Infeasible sex–disease combinations (e.g., male breast cancer) were removed. Both arms received a clinical letter signed by the medical director of the hereditary cancer genetics program at Rutgers Cancer Institute. Participants assigned to the EUC arm were sent the letter via email, postal mail, and/or patient portal informing them of their and their relatives’ potential hereditary cancer risk. The letter emphasized the participant’s eligibility for GT, recommended scheduling a GC appointment, and included contact information for the GC clinic, including a link to the website. In contrast, DHG participants were granted access to Alex (DHG), and after completing the required genetic education modules, they could proceed directly to making a GT decision. Participants completed post-intervention surveys 1 and 6 months after receiving the EUC letter or engaging with the DHG platform. Participants received a $50 gift card for each completed survey (baseline, 1- and 6-month post-intervention surveys), and an additional $30 for completing both follow-up (1- and 6-month) surveys, for a potential total of $180 in gift cards.

#### Measures

The baseline survey assessed demographics and clinical characteristics. Both baseline and post-intervention surveys assessed GT knowledge, a 10-item knowledge index that measures a patient’s understanding of inherited cancer concepts covered during a pretest GC session (Cronbach’s alpha = 0.69) (Cragun et al. 2020). Additionally, the post-intervention assessment collected data on (1) GC and GT uptake measured by the proportion of participants in each study arm who received GC and/or GT, verified by EHR and lab results; (2) Decisional Conflict Scale (O’Connor, 1993), a 16-item measure assessing how informed, clear, supported, autonomous, and confident a person feels in making a GT decision, and the degree of uncertainty they experience (Cronbach’s alpha = 0.88); (3) Decisional Regret (Brehaut et al. 2003), a 5-item measure assessing the level of distress or remorse regarding their decision (Cronbach’s alpha equals 0.89); and (4) DHG acceptability for DHG arm participants using the CUQ (Holmes et al. 2019).

#### Data analysis

Descriptive statistics were calculated for sociodemographic and clinical characteristics. Feasibility was determined by calculating the cooperation rate (number enrolled divided by number contacted and screened eligible on the internet eligibility survey) and retention rate (number who completed the follow-up survey divided by number enrolled). Average CUQ scores (median and interquartile range (IQR)) were calculated to assess the acceptability of the chatbot for those in the DHG arm. Chi-square tests (and Fisher’s exact test, when appropriate) were used to evaluate GC and GT uptake between EUC and DHG patients 6 months post-intervention. The Wilcoxon matched-pairs signed-rank test was used to compare pre- and post-intervention GT knowledge for all patients and stratified by study arm. The Wilcoxon rank sum test was used to compare decisional conflict, decision regret, and decision satisfaction between the EUC and DHG arms. The non-parametric Wilcoxon tests were used due to the skewness of the data. All analyses were conducted using an intention-to-treat approach.

## Results

### User and usability testing

Fourteen participants participated in the interviews: 6 for user and 8 for usability (eFigure 1, Supplement). The majority were non-Hispanic White (64.3%), female (78.6%), and had a prior history of breast cancer (78.6%), GC (57.1%), and GT (78.6%). The mean age of these participants was 55.9 (SD = 10.8) years. Sociodemographic and clinical characteristics are detailed in Table 1.

### User testing experience

The illustrative quotes are presented in Table 2.

**Table 1:** Demographics and characteristics of participants

Characteristics	User testers (*n* = 8)	Usability testers (*n* = 6)	Pilot trial participants (*n* = 28)	
			EUC (*n* = 13)	DHG (*n* = 15)
Age (years)				
Mean (SD)	51.1 (±10.8)	62.3 (±7.5)	54.1 (±15.4)	61.0 (±14.3)
Gender, n (%)				
Male	1 (12.5)	2 (33.3)	7 (53.9)	7 (46.7)
Female	7 (87.5)	4 (66.7)	6 (46.1)	8 (53.3)
Race, n (%)				
White	4 (50.0)	5(83.3)	8 (61.5)	12 (80.0)
Black or African American	2 (25.0)	0 (0.0)	1 (7.7)	1 (6.7)
Other^a^	2 (25.0)	1 (16.7)	4 (30.8)	2 (13.3)
Ethnicity, n (%)				
Hispanic or Latino	0 (0.0)	0 (0.0)	4 (30.8)	4 (26.7)
Not Hispanic or Latino	8 (100)	6 (100.0)	9 (69.2)	11 (73.3)
Marital Status, n (%)				
Single/ Separated/Divorced/Widowed	5 (62.5)	3 (50.0)	7 (53.8)	6 (40.0)
Married/Civil Union or living together	3 (37.5)	3 (50.0)	6 (46.2)	9 (60.0)
Employment Status, n (%)				
Full-time	4 (50.0)	3 (50.0)	8 (61.5)	5 (33.3)
Part-time	0 (0.0)	0 (0.0)	0 (0.0)	2 (13.3)
Retired	1 (12.5)	1 (33.3)	2 (15.4)	6 (40.0)
Other	3 (37.5)	1 (16.7)	3 (23.1)	2 (13.3)
Household Income				
Less than 35,000	2 (25.0)	0 (0.0)	4 (30.8)	3 (20.0)
$35,000-74,999	2 (25.0)	0 (0.0)	2 (15.4)	3 (20.0)
$75,000-99,999	0 (0.0)	3 (50.0)	1 (7.7)	2 (13.3)
$100,000 or more	4 (50.0)	3 (50.0)	6 (46.2)	7 (46.7)
Cancer Diagnosis, n (%)				
Breast	7 (87.5)	4 (66.7)	0 (0.0)	0 (0.0)
Ovarian	0 (0.0)	0 (0.0)	3 (23.1)	4 (26.7)
Colorectal	1 (12.5)^b^	0 (0.0)	2 (15.4)	3 (20.0)
Prostate	0 (0.0)	2 (33.3)	2 (15.4)	3 (20.0)
Endometrial	0 (0.0)	0 (0.0)	4 (30.8)	3 (20.0)
Pancreatic	0 (0.0)	0 (0.0)	2 (15.4)	2 (13.3)
History of Genetic Counseling, n (%)			-	-
Yes	5 (62.5)	3 (50.0)		
No	1 (12.5)	3 (50.0)		
Unsure	2 (25.0)	0 (0.0)		
History of Genetic Testing, n (%)				
Yes	8 (100.0)	3 (50.0)		
No	0 (0.0)	3 (50.0)		
Unsure	0 (0.0)	0 (0.0)		
Chatbot Usability Questionnaire			-	
Median (IQR)	75 (±25)	81.3 (±17.2)		70.3 (±12.5)^c^

^a^Includes Asian, Native American/Alaskan Native, and Multiple races

^b^Patient also has endometrial cancer

^c^Only the RA patient engaged with the DHG

#### Theme 1: User trust and engagement

This theme focuses on the interaction between the user and the DHG, Alex. It encompasses the perceived reliability of the DHG in providing accurate information, the user’s trust in Alex, and how Alex’s persona influences engagement. These factors contribute to how users perceive Alex and its usefulness in decision-making.

**Table 2:** Digital health guide prototype user and usability testing experience

Themes	Components	Description	Illustrative quotations
User Trust and Engagement	Chatbot Persona	The health guide’s personality and tone influence user engagement and perception. A more human-like, approachable persona can create a better connection with users.	*“I feel it’s worded in a way that’s respectful and informative. It does feel like a person is asking you versus a chat.* *“I’m disappointed with Alex’s voice. Not the voice I thought, monotone.” * *“He’s a nice gentleman.” * *“It sounded too robotic.” *
	Trustworthiness of the Health Guide	Users need to trust the health guide’s security and the credibility of the health information provided. Privacy and data security are key considerations.	*“I like that it’s sponsored by the Rutgers Cancer Institute because there’s credibility behind it…there’s a backstory there, and you know where your information is going.” * *“I am very confident [login is secure] because I was given my personal username, and it then asked me to create my password. So I believe, even if they have the username, the password is unique to me.” * *“I feel confident that they are going to put the safety measures in place to keep people’s identity, health information, all of these private things, private.” * *“As I’ve been using it and going through the process, I’ve gained confidence that it’s more secure.” *
	Dissemination of Information	The health guide’s ability to help users share and discuss genetic testing information with others, such as family members, plays a key role in the user experience.	*“If I wanted to encourage my other family members to get screening, I would definitely show them this.”* *“It might be hard for people to talk about, so it helps you start the conversation.” * *“If it was bringing this to a doctor in order for them to know that I need to do genetic testing, I would definitely bring this entire thing.” *
Informed Decision-Making and User Empowerment	Impact on Users to Make a GT Decision	The health guide influences how users make decisions about genetic testing, including reducing decision-making anxiety and providing clarity.	*“[Alex] just kept on saying do you think you should be tested and all this stuff. I wouldn’t have struggled that much on whether I should get tested or not.”* *“If they’re trying to get to the heart of the matter, they probably want to get to the point sooner.” * *“I would think that if there's any interest in health or themselves, it would be a nice, quick, easy way to see what's going on and to get some results.” * *“I think it's a good option to help people decide if they're ready to go through with it or not. If they tend to be a very anxious person, that might lead them to go to their healthcare provider instead of doing it on their own. If they are levelheaded, and this is something they've already watched family members go through, it might make a speedier decision on the testing and allow them to go through it with little to no aggravation.” *
	Address Personal Barriers and Facilitators	The health guide helps users overcome barriers to genetic testing, such as time, medical knowledge, and access to healthcare. It empowers users by providing autonomy and flexibility.	*“I felt like it was very neutral, provided information without being emotional, drama-free, to the point, and it allowed patients a way to start without having to wait two months to get in to see their doctor to have them either - one, rush them through it or the opposite response of 'we don't need that'. It gives the patient a little more autonomy and decision-making in pursuing information about themselves.” * *“This is good, based on what the patient or person is looking for they can basically scroll over to those type of questions and they have more information pertaining to the related topic that they're looking at. I like this one - what type of questions you should ask your doctor, they have a PDF here, so if you are really in that situation, you don't know what to ask, so you have some help available to you.” * *“I was impressed with the fact that it is concise. It was screening. And I like the kit because a lot of people don't have time or can't get appointments, so it can expedite results and it can expedite usability and put them at ease whether or not they're at risk.” * *“I think that was pretty simple. I think the only thing is - but this is reality - I think the minute anybody hears if there's any fees, I think that throws everybody. But that is just the reality. It depends on if you have a copay or what you've met in your deductible. I just feel like when people start to hear that, I just think people move away from moving forward. But that is not anything to about the actual system itself; you have to call attention to it because somebody may get charged.” *
	Meeting Individual Needs	The health guide adapts to meet users’ specific needs, such as physical limitations, medical knowledge, or understanding of health-related terms.	*“I have Raynaud’s, and sometimes I cannot feel my fingers. Sometimes, I can’t use a touch screen because my fingers have no blood to tell the screen to do something, so for me, voice activation is beneficial.” * *“I think a lot of people are laymen and may not understand the questions.” * *“It was pretty simple, easily understandable. I do not have any medical background, so it’s hard for me to understand the questions, but I was able to read through it.” *
Ease of use and cognitive load	User Knowledge Burden	The health guide’s ability to accommodate users with limited knowledge of genetic testing and family health history. It reduces the burden of having to know specific information.	*“I like that it’s not cut and dry, black and white…it’s not punitive if you don’t know everyone in your family and what they had.” * *“There's a lot of holes in my own family history and if I'm able to get through [the platform], I think probably other people should be able to.” * *“Keeping in mind that people don't always know. Like if I'm not in touch with my cousins, I don’t know what they may or may not have been struggling with. But since it gives you the opportunity to say I don't know, I think it's good just the way it is.” *
	Clarity of Content and Navigation	The health guide’s content is clear, and its navigation is simple and user-friendly. It helps reduce cognitive load by making information easy to process.	*“It was easy to navigate; it used everyday language and was more expedient than trying to get appointments.” * *“I think it was very clear and informative.” * *“The flow is great; I think it’s simple.” * *“I think it was put together well.” * *“I think it’s very informative, very helpful and, yeah, relatively easy to circumvent.” *
	Sensitivity	The health guide’s ability to convey sensitive information (e.g., genetic test results) in a compassionate and non-threatening manner.	*“It ’s just kind of giving us a hard pill in the softest way possible, so I do appreciate that because we need this information.” * *“This is good. I was hoping that you weren't telling me that I'm positive in the email. I would prefer, like you said please contact us. Because I would want it more personal.” * *“I would not react with fear to that because it says 'it looks like', and it talks about 'developing' [cancer] rather than just 'you have it'.” *

##### *Chatbot persona*

User reactions to Alex’s persona were mixed, particularly regarding its voice and tone. Some testers appreciated the voice’s clarity, professionalism, and respectfulness, while others found it monotonous and robotic, which detracted from the user experience. The mechanical tone was seen as less engaging, with some users noting it made interactions feel tedious. A more human-like, relatable persona was suggested to improve the DHG’s quality.

##### *Trustworthiness of the DHG*

Some users raised concerns about privacy and security, particularly regarding third parties’ potential exposure and misuse of their data. Despite these concerns, users’ trust in the DHG was bolstered by its association with reputable institutions (Rutgers University and the Rutgers Cancer Institute), which increased their confidence in its security. Additionally, users preferred accessing the DHG hosted by trusted medical settings rather than independently online. While many users felt secure using the platform after learning about its security measures, some remained cautious due to past experiences with identity theft.

##### *Dissemination of information*

Users expressed high satisfaction and trust in Alex for facilitating GT discussions and sharing information with family and healthcare providers. They appreciated Alex’s ability to simplify the dissemination of information, making it easier to share education and results with family members and doctors. Most viewed the DHG as valuable for initiating sensitive conversations about GT and were eager to recommend it to others.

#### Theme 2: Informed decision-making and user empowerment

This theme captures Alex’s role in assisting users and users in making informed decisions about GT. It addresses how the tool helps users overcome personal barriers, supports users in decision-making, and tailors the experience to individual needs, ultimately empowering users to make decisions that align with their personal circumstances.

##### *Impact on users to make a GT Decision*

Testers had mixed opinions on Alex’s ability to influence users in making GT decisions. Some found the initial prototype’s direct approach helpful, while others felt it was too forceful, prompting users to consider testing while they are still trying to understand their risk of hereditary cancer. Videos featuring personal testimonials from survivors and family members resonated with many, making the content more relatable and impactful. However, some testers felt Alex’s approach might not engage those with no prior interest in GT, especially if they lacked a personal connection to the issue. Testers suggested that a more streamlined, interactive approach could better motivate reluctant patients.

##### *Address personal barriers and facilitators*

Users recognized that Alex could reduce personal barriers to GT by creating an accessible information platform that users valued for its convenience and privacy, particularly among those who felt anxious about face-to-face interactions with healthcare professionals. Participants acknowledged Alex’s ability to help navigate common concerns like lack of knowledge, insurance coverage, and financial costs.

##### *Meeting individual needs*

Users emphasized the need for the DHG to accommodate various literacy levels, cultural backgrounds, and physical abilities. Features like voice activation were praised for users with physical challenges, such as difficulty using touchscreens. Concerns were raised about attention span and reading comprehension, particularly among older users or those unfamiliar with lengthy reading. Suggestions included visual aids, like graphs or simple illustrations, to clarify complex information for users without a scientific background. The speed of information delivery was another concern. Users noted that rapid message delivery could be difficult for slower readers, so allowing users to control the pace of the interaction was recommended. Simple, clear language was appreciated, but overly technical terms were considered a barrier for some users.

#### Theme 3: Ease of use and cognitive load

This theme focuses on the DHG’s usability and the user’s mental effort. It includes how clear and navigable the platform is, how the DHG manages the user’s cognitive load, and how sensitive it is to the user’s emotional or informational needs.

##### *User knowledge burden*

The DHG was also well-received for accommodating testers with limited knowledge of their family history or medical background. Some concerns were raised about testers without any knowledge of their family’s health history, but the DHG’s flexibility was seen as a key facilitator. Additionally, while some testers found the medical and genetic terminology overwhelming, they appreciated Alex’s ability to help them navigate the process without requiring expertise. The DHG’s flexibility and user-friendly design made GT more accessible, even for those with limited medical knowledge or family health details.

##### *Clarity of content and navigation*

Testers generally found the DHG’s content and navigation clear, noting its simplicity, logical flow, and ease of use. However, some testers experienced confusion due to unclear prompts, unexpected transitions, and certain design elements, such as pop-ups or buttons that were not immediately intuitive.

##### *Sensitivity*

Alex’s approach to delivering sensitive information, such as GT results, received mixed but largely positive feedback. Many users appreciated the calming, supportive tone, which helped alleviate anxiety during emotionally intense experiences. However, some testers suggested that more personal interaction might be necessary when delivering serious or life-changing information. Some testers felt all results should be delivered by a human, while others believed this concern could be mitigated by enhancing Alex’s empathy and communication style.

### Usability

The usability assessment findings were categorized into three areas: error occurrence (instances when the platform did not meet operational expectations), researcher intervention (instances when the researcher needed to step in to guide the user through the platform’s flow), and CUQ score.

#### *Error occurrence (technical competence)*

Ten out of 14 testers experienced at least one technical error during their interactions with the platform. Tasks that were particularly prone to errors included “Pedigree/Family Tree,” “Genetic Testing Choice,” and the “Tabs (Chat/Questionnaire) (eTable 3 and eFigure 3, Supplement).

#### *Researcher intervention (intuitiveness of navigation)*

Eight out of the 14 testers required researcher intervention due to navigation challenges. Tasks such as “Account Creation,” “Onboarding,” and sections involving cancer family history and cancer family risk questionnaires often caused confusion and required assistance (eTable 3 and eFigure 4, Supplement).

#### *CUQ score*

The average CUQ score for the user testers was 75 (IQR = 25), and 81.3 (IQR = 17.2) for usability participants, exceeding the acceptability benchmark of 68.

#### Improvements to the DHG based on user and usability testing feedback

**Fig. 2:**
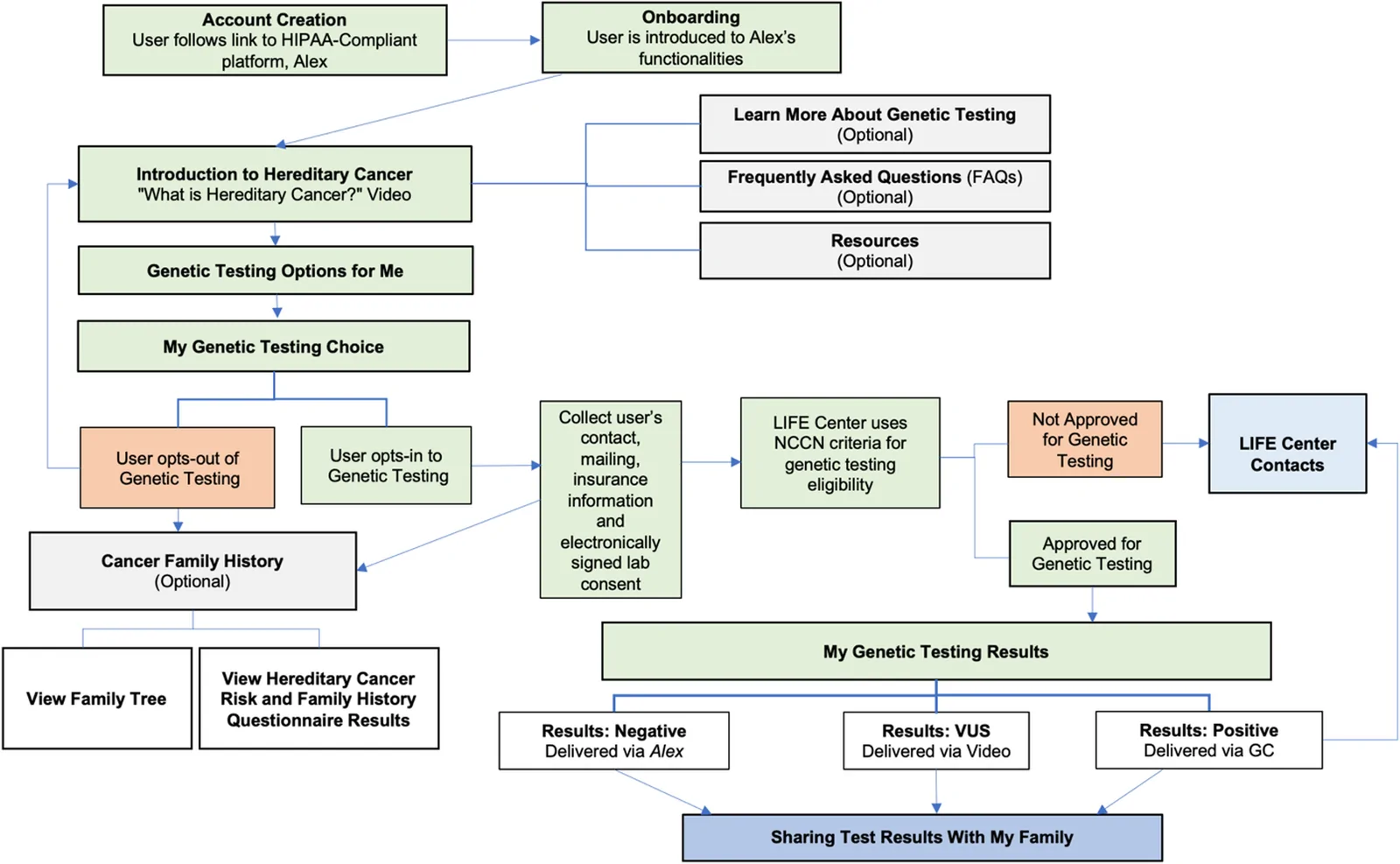
Digital Health Guide prototype user journey after user and usability testing

User feedback led to several improvements aimed at enhancing the functionality and user experience of the DHG (Fig. 2). Users suggested clearer, more direct language, especially in health-related contexts. For example, the DHG now states that GT is “highly recommended” based on risk level instead of asking if users “want” or “are worried” about testing. Alex was refined to an 8th -grade or lower literacy level for each module. Alex’s responses were shortened to improve focus and engagement. Visual prompts and clearer button labels, like “My Menu,” were added to enhance navigation.

Aesthetic updates included reducing white space and introducing color and imagery to create a more visually engaging platform. Alex’s icon was integrated into the Cancer Family History Questionnaire to break up text and strengthen the user’s connection to Alex as a guide through the GT process. The Family Tree design was improved with a larger font, a relocated legend, and a stronger contrast between elements and the light blue background. The DHG’s backdrop and header color were changed to a more stimulating blue, and the arrows on the FAQ and Learn More About Genetic Testing carousels were enlarged for better visibility. The menu was restructured to a drop-down format with clearer spacing between options. Timestamps were added to educational videos to indicate their length, and the DHG now autofits to all screen sizes, including a progress bar and animated icons to enhance user interaction.

Usability improvements were also made based on user feedback. The login process was simplified with step-by-step instructions and reassurances about security. A new button was added to the menu for easier access to the Knowledge Base, and the microphone feature was refined to reduce unnecessary voice narration. Voice chat responses were streamlined to focus on essential information, and the voice itself was upgraded to sound clearer, more humanlike, and consistent across browsers. The speed of Voice Chat and text notifications was slowed to improve clarity and reduce robotic tones.

Content revisions were made to address user needs more effectively. The FAQ section was expanded to include answers to questions such as, “What if I can’t find information about my family history?” A new “Quick Reply” option allowed users to skip extra content and go directly to the Cancer Family History Questionnaire. For users receiving their hereditary cancer risk statement, Alex now offers resources to manage stress, including contact details for a genetic counselor. To address the comment that the DHG may appear forceful and encouraged users to consider testing while still trying to understand, a new option was added to the Genetic Testing Choice process for users who prefer additional time to process the information before making a decision. In addition, the DHG now provides explicit guidance on how users can contact a cancer genetic counselor at the LIFE center if they are uncertain or have further questions or concerns while considering their options.

The DHG was refined to be more streamlined and interactive by adding a progress bar and an animated “send message” icon, which made Alex’s interface feel more dynamic and responsive. Additional Quick Reply options were incorporated, allowing users to skip or advance sections, thereby creating a more streamlined flow that directly addressed testers’ feedback requesting a shorter and more straightforward experience. The Cancer Family History Questionnaire was made optional and repositioned to the end of the flow, following the user’s GT decision. Visual enhancements, including updates to colors, imagery, and layout, were also implemented to sustain engagement and make the platform feel more legitimate and interactive. Revisions also focused on fostering more human-like and supportive interactions within the platform. When delivering the hereditary cancer result, Alex now provides contact information for a cancer genetic counselor. We also added a new Resources page featuring external supports/websites, where patients can connect to additional resources/information, including empowerment groups like Facing Our Risk of Cancer Empowerment (FORCE). These additions acknowledge the potential emotional impact of receiving serious information. Alex’s written tone, speech speed, and voice for the “Voice Chat” feature were also changed to sound less robotic and more conversational, and positive affirmations were integrated throughout the Cancer Family History Questionnaire to offer encouragement and emotional support.

Additional content updates included a more informative introductory line to clarify the DHG’s purpose and revisions to video and form elements. The Readiness questionnaire was shortened based on feedback, reducing it from four to three questions and clarifying the language for better understanding. The “importance” sequence was updated to explain the ability to select, deselect, and choose multiple response options. The response option “I don’t have any barriers” was rephrased to “I don’t have any barriers to moving forward with genetic testing.” A technical issue with the Introductory Video volume was also resolved. The Risk Assessment module was removed to streamline the flow, and the Cancer Family History Questionnaire was made optional and relocated to appear after the user made their GT decision.

**Table 3:** Randomized pilot trial results for decisional conflict, decisional regret, and GT knowledge

Measures	Possible range	Mean ± SD		MD ± SD	z^c^
		EUC arm	DHG arm		
Decisional conflict	0-100	53.25 ± 23.66	33.37 ± 21.09	19.87 ± 8.22	2.234^a^
Decisional regret	0-100	37.08 ± 17.38	17.5 ± 16.50	19.58 ± 6.65	2.547^a^
GT Knowledge	0-10	Pre-intervention	Post-intervention		
All patients		3.19 ± 2.52	3.53 ± 2.25	0.62 ± 1.76	-1.782^b^
EUC		2.92 ± 2.11	3.90 ± 2.43	0.36 ± 1.75	-1.394^b^
DHG		3.43 ± 1.65	4.00 ± 1.75	0.57 ± 1.74	-1.178^b^

^a^Wilcoxon rank sum test

^b^Wilcoxon signed rank test

^C^Test statistics (Z) are reported without p values, consistent with the exploratory and underpowered nature of this feasibility trial

Some suggestions were not implemented as they either conflicted with other users’ feedback, did not align with the DHG platform’s overall objectives, or were beyond the scope of what the chatbot developer could implement because of the research budget constraints. These included changes to the Family Tree design (such as replacing shapes with male and female symbols), adding two-factor verification (e.g., Duo) for login, changing the CATALYST logo and color scheme, and adding multilingual support. These changes will be incorporated into the DHG, that will be tested in a planned definitive randomized controlled trial.

### Feasibility randomized trial

**Fig. 3:**
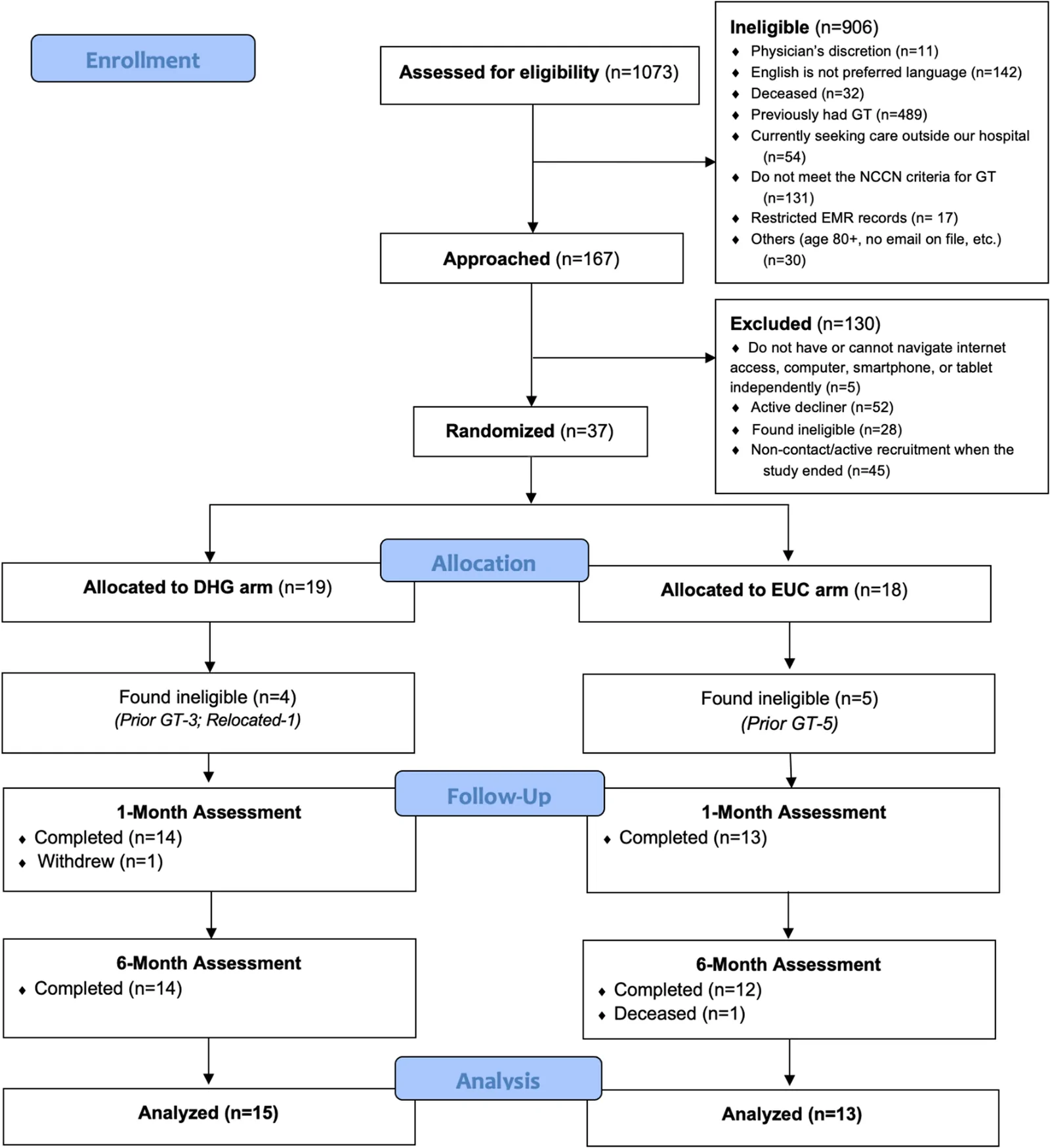
CONSORT flow diagram for the pilot study

The response rate among those who were contacted and deemed eligible was 77.1% (37/48). Of the 37 patients randomly assigned to the 2 arms, 9 were found to be ineligible after randomization (9 had prior GT, while 1 relocated to another state). Thus, 28 patients (13 in EUC, 15 in DHG) were included in the final analysis (Fig. 3). Demographic and clinical characteristics of the participants are displayed in Table 1. The one-month retention rate was 96.4% (27/28), the six-month retention rate was 92.9% (26/28).

The median CUQ score for the DHG arm was 70.3 (IQR = 12.5), indicating good acceptability (Table 1). A review of the DHG’s chat logs did not find any cases of incorrect information or hallucinations (instances in which the chatbot confidently returns inaccurate or out-of-scope content rather than invoking the programmed fallback or CGC/study team escalation). Of the 15 participants randomized to the DHG, 14/15 (93.3%) created an account on the platform, 13/15 (86.7%) met the engagement threshold (reached the GT choice module) and made a GT decision, and 11/15 (73.3%) requested a GT kit.

Almost three-quarters (73.3% (11/15)) of DHG arm participants had GT compared to only 7.7% (1/13) in the EUC arm. In the EUC arm, the only patient to request pretest GC was the one patient who completed GT. No patients in the DHG arm requested GC. The participant in the EUC arm who requested GT received a VUS result, whereas among the 11 participants in the DHG arm who requested GT, 4 received VUS results and 7 received negative results.

Six months after the intervention, DHG participants had appreciably lower decision conflict (33.37 ± 21.09) and decision regret (17.5 ± 16.50) than the EUC participants (53.25 ± 23.66 and 37.08 ± 17.38, respectively) (Table 3). However, no notable difference in GT knowledge was observed between the arms.

## Discussion

Our study provides valuable insights into the development, acceptability, usability, feasibility, and preliminary efficacy of a chatbot designed to facilitate GT uptake among cancer patients who met national criteria for genetic testing. Community and patient engagement provided critical input into the study design. As presented earlier, we refined the intervention before the feasibility trial by incorporating feedback from focus groups, users, and usability participants. The DHG was found to be feasible and acceptable among pilot trial participants. Participants reported that the DHG effectively addressed their GC and GT needs. Overall, the participants described the chatbot as engaging, informative, and well-designed. The high user satisfaction aligns with previous research indicating that chatbots and web-based technologies can effectively deliver and help navigate genetic information (Wah 2025). The DHG’s acceptability was supported by its CUQ score above the benchmark of 68, its privacy and convenience of use, its Rutgers affiliation, and the platform’s compliance with HIPAA standards. In addition, a cooperation rate of 77.1% and retention rates of 96.4% (1-month) and 92.9% (6-month) in the pilot trial support the fact that the intervention is highly feasible. 

Based on users’ feedback, several improvements were made, including clearer language, 8th grade (or lower) literacy level, enhanced navigation, improved content (e.g., expanding FAQs), design (e.g., adding icons for insurance uploads), functionality (e.g., refining pedigree display and video sound quality), and a stronger emphasis on data security, privacy and confidentiality. These adjustments were aimed at improving the overall user experience. By integrating these patient/user-driven enhancements, the DHG may be better positioned to meet both the informational and emotional needs of diverse cancer survivors, further reinforcing the importance of community and patient inputs to ensure that chatbots are responsive to the tailored needs and concerns of cancer patients and their communities at large (Lawson Mclean and Hristidis 2025; Ma et al. 2024). 

Research has shown that automated tools such as chatbots can achieve GT rates comparable to or even higher than the traditional GC referral and GT process (Al-Hilli et al. 2023; Kaphingst et al. 2024; Webster et al. 2023). The high rate of GT uptake among the DHG participants in our study suggests that it is a viable alternative to in-person GC to minimize barriers to both genetic education and testing while streamlining care and expanding access to more cancer patients for timely genetic education and guideline-based testing. This increased reach of GT may be explained by DHG’s ability to address multiple barriers, including travel distance to GC services, limited availability of GC staff, and long appointment wait times. This finding further underscores the DHG’s potential in addressing critical gaps in GT, thereby supporting broader implementation in the clinical oncology care continuum (Schmidlen et al. 2022a). Given that the DHG not only offers genetic education and counseling but also facilitates the GT process and relays GT results, we explored GT uptake as a direct assessment of how effectively the DHG bridges the gap between counseling and GT uptake and completion compared to usual care. We also prioritized GT uptake as the key outcome given persistent underutilization of germline testing among eligible cancer patients, making uptake a clinically meaningful and implementation-relevant endpoint for a pilot intervention aimed at reducing barriers to testing.

Additionally, participants randomized to the DHG arm showed significantly lower levels of decisional conflict and decision regret regarding the decision to pursue GT. Only one of the EUC participants opted to pursue GT (and pretest GC) during this study, despite being provided with a clinical letter emphasizing its utility and a referral for cancer GC and GT. The DHG participants received considerable additional salient information, both through Alex and via the receipt of test results and follow-up recommendations, which alone could account for their better decisional outcomes. In addition, 11 participants in the DHG arm received either negative or VUS results, both of which were generally reassuring for them and their family members, and this likely contributed to their more favorable decisional outcomes. Conversely, EUC participants were informed of their and their relatives’ potential risk and referred to the cancer genetics clinic, but received little additional information, with only one proceeding to GC and GT. This increased ambiguity about their risk may have contributed to more negative decisional outcomes, and it is also possible that some were still considering testing. Overall, DHG supported patients in making decisions, and they were largely satisfied with the choices they made, even among those who declined GT, as these decisions were made in the context of sufficient information. These findings align closely with the ODSF (Hoefel et al. 2020a, b; Stacey et al. 2020). Additionally, DHG participants were encouraged to continue engaging with the platform until they had viewed all the genetics education material. This prompting may have encouraged them to complete all the modules before making a decision, whereas they might otherwise have made an uninformed GT decision with incomplete information or chosen not to test at all. Thus, the easy access to and content of the genetic education may have contributed to lower decisional conflict and decision regret among DHG arm participants. The similar level of knowledge observed between participants in both arms post-intervention could be due to the possibility that participants looked up answers to these questions on their own after completing the baseline survey or receiving the clinical letter.

In our study, the DHG’s chat logs did not find any cases of incorrect information or hallucinations. The chatbot was designed to minimize misinformation and hallucinations by refining/limiting the knowledge base (Alex’s pool of information) and restructuring the instruction prompt that guides how Alex delivers information (e.g., tone, literacy level, “I cannot answer that, please contact study staff”). These strategies together tightened Alex’s restrictions on what it can answer and streamlined the information provided to users, reducing the risk of inaccurate or overly complex responses.

Our findings showing acceptability, feasibility, and preliminary efficacy provide strong justification for a larger randomized controlled trial to determine the efficacy of our approach (proactive clinical outreach and streamlined virtual genetic care delivery model) before broad dissemination. We have planned a multi-site RCT in two large health systems with over 19 academic and clinical sites to assess the efficacy and scalability potential beyond our health systems. In preparation for the planned multi-site RCT, we have developed and are currently testing family sharing of GT results and cascade testing modules for PV-positive probands and their at-risk relatives. This approach can leverage the multiplier effect and substantially increase the identification of mutation carriers and reduce preventable cancer burden.

This study has several limitations. The small sample size, typical of a feasibility trial, limits the generalizability of the findings, warranting a larger, definitive trial to establish efficacy. Additionally, there may be recruitment and retention biases (e.g., overrepresentation of already motivated individuals and underrepresentation of underserved populations) that might impact the results. Using an equity-enhanced implementation science framework (Shelton and Brownson 2024), a larger trial could help assess fairness in need, reach, and outcomes to design tailored approaches (e.g., cultural and linguistic) for underserved populations. Moreover, researcher interactions during usability testing may have influenced user experiences, highlighting the need for future studies to assess the platform in more natural, less guided settings, such as the pilot trial. A larger, more diverse sample to establish efficacy would enhance generalizability. Another limitation was the limited diversity in the user/usability testing participants by race and cancer type. Lastly, though the DHG is a useful tool that can streamline genetic care delivery and increase access to education and GT, it is not designed to bypass regulatory requirements, as a medical professional still needs to place the GT order and help obtain health insurance authorization.

Despite these limitations, our study has several strengths, including a participatory research approach and responsiveness to patients’ needs and preferences. A user-centered approach was central to the DHG design, ensuring it was evaluated and refined based on user feedback. The methodological rigor was further strengthened by using the Framework Method for qualitative data analysis, which facilitated systematic interpretation of the data and ensured intercoder reliability (Cohen’s κ = 0.96). These elements and the diversity of user feedback from individuals with varying demographics provided comprehensive insights into the platform’s performance. The randomization, coupled with adherence to the intention-to-treat principle, enhanced the internal validity of our findings and minimized potential bias.

In conclusion, we found our DHG to be feasible and acceptable, and a promising strategy for expanding access to the reach of education and GT by streamlining care and reducing barriers for cancer survivors and their relatives. By supporting informed decision-making and removing key access barriers, the DHG helped patients make informed choices about GT. Further testing in a definitive RCT is warranted to determine efficacy and implementation potential.

## Supplementary Information

Below is the link to the electronic supplementary material.

Supplementary Material 1

## Data Availability

Deidentified data from this study are not available in a public archive under the current consent form.

## Declarations

This study was performed in line with the principles of the Declaration of Helsinki. Approval was granted by the Institutional Review Board of Rutgers, the State University of New Jersey (#Pro2023000964). Written informed consent was obtained from all individual participants included in the study.
