# Supplementary material for: Development and feasibility testing of a conversational chatbot supporting genetic education and testing for hereditary cancer
Source: J Community Genet. 2026 Jun 22;17(3):49. doi: 10.1007/s12687-026-00873-z (PMC13287281; doi:10.1007/s12687-026-00873-z)
Supplement: Supplementary file 1 — Supplementary Material 1 [file 12687_2026_873_MOESM1_ESM.docx]

**SUPPLEMENTARY INFORMATION**

**eFigure 1.** Flow diagram for the user and usability testing.

**eFigure 2.** NCCN criteria for germline testing for selected cancer patients.

**eFigure 3.** Error occurrence log for the catalyst prototype found during user and usability testing.

**eFigure 4.** Researcher intervention log during user and usability testing.

**eTable 1.** Sociodemographic and clinical characteristics of focus group patients.

**eTable 2.** User testing interview guide

**eTable 3.** Error occurrence and researcher intervention during prototype usability testing


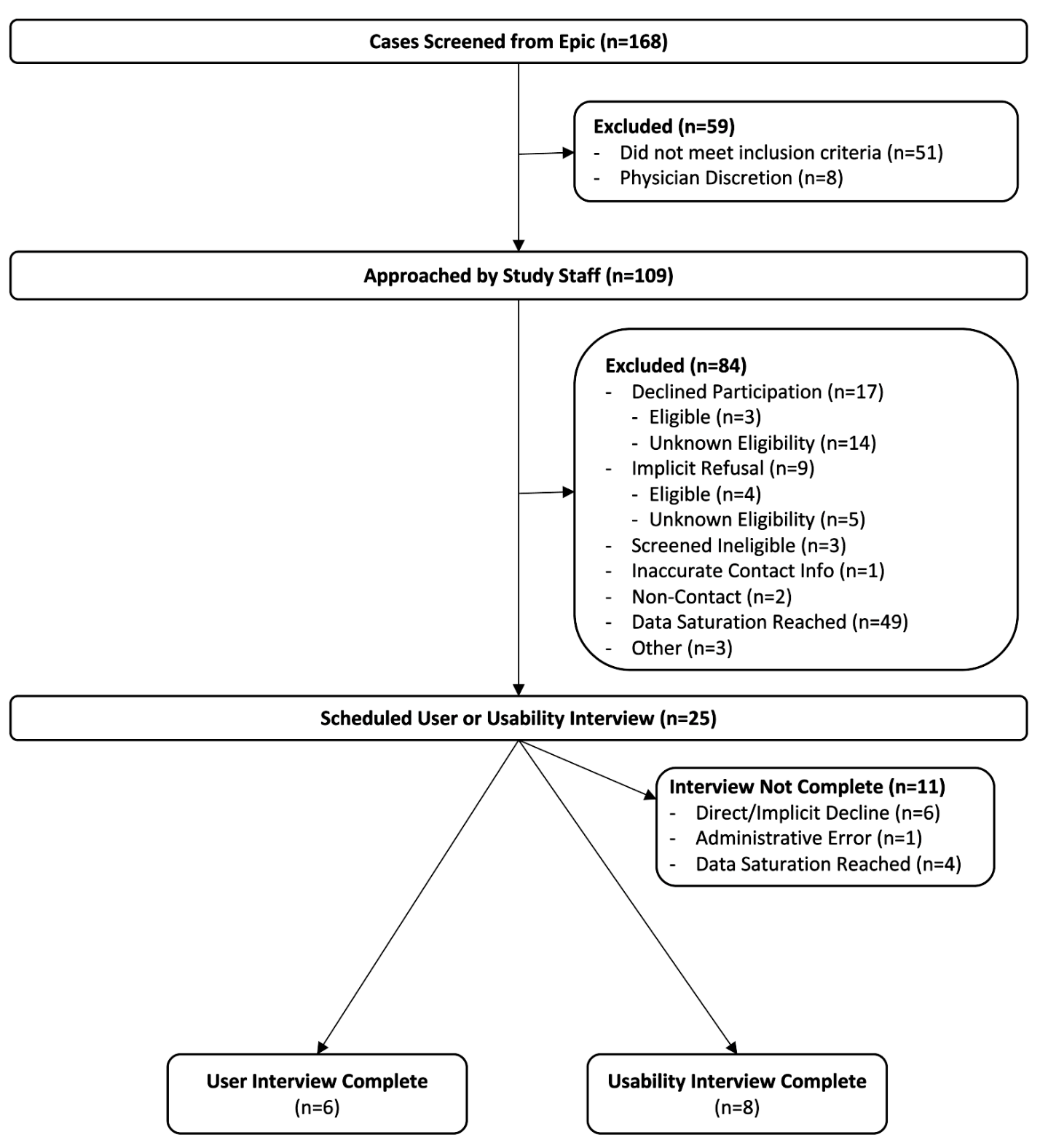


**eFigure 2.** Flow diagram for the user and usability testing


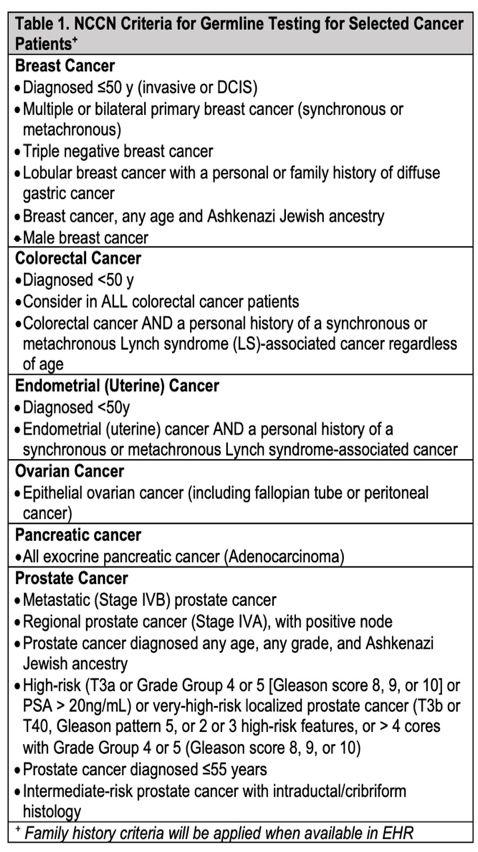


**eFigure 1:** NCCN criteria for germline testing for selected cancer patients (NCCN, 2025a, 2025b)


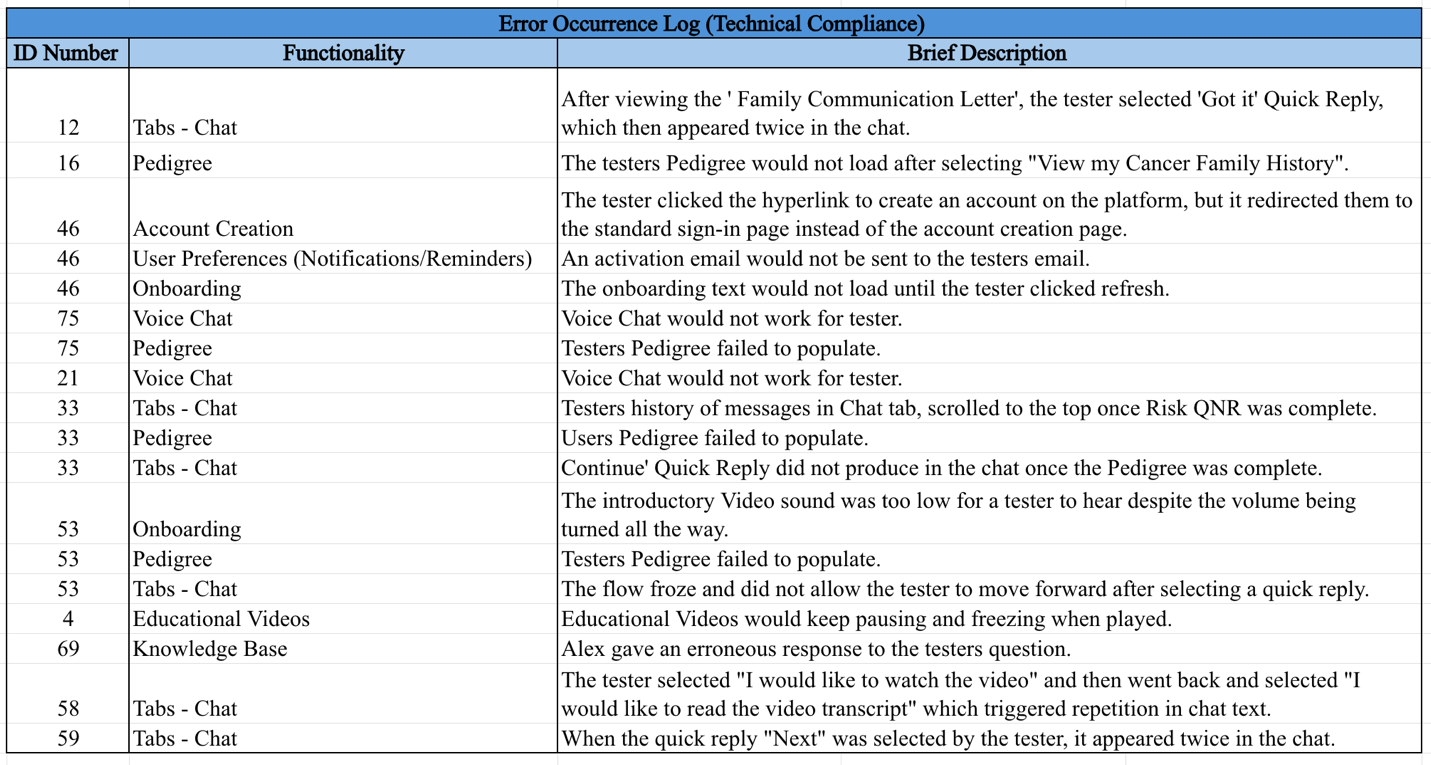


**eFigure 3.** Error occurrence log for the catalyst prototype found during user and usability testing


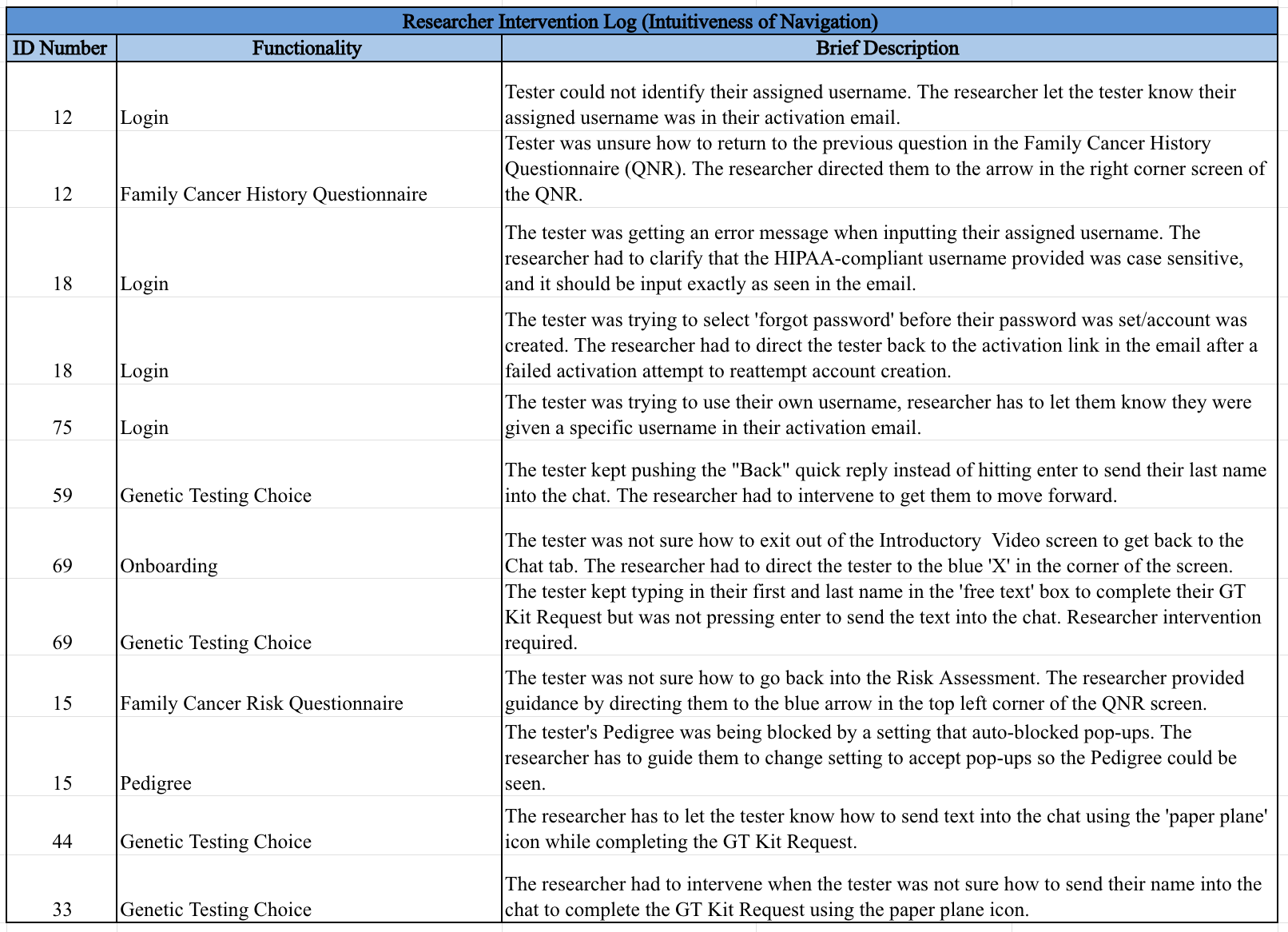


**eFigure 4.** Researcher intervention log during user and usability testing

**eTable 1: Sociodemographic and clinical characteristics of focus group patients**

| **Characteristics** | **n(%)**  **51 (100)** |
| --- | --- |
| **Participant type**  Mutation carrier  Cancer patients  Relatives of cancer patients | 24  18  9 |
| **Age**  Mean (SD) in years | 47.3 (±17.29) |
| **Sex**  Female  Male | 36 (70.5)  15 (29.5) |
| **Race**  White  Black/African American  Others^a^ | 28 (54.9)  14 (27.5)  9 (17.6) |
| **Ethnicity**  Hispanic  Non-Hispanic | 12 (23.5)  39 (76.5) |
| **Ashkenazi Jewish ancestry**  Yes  No | 14 (27.5)  37 (72.5) |
| **High level of education**  High school diploma or lower  College graduates  Graduate school or professional | 12 (23.5)  23 (45.1)  16 (31.4) |
| **Marital status**  Single  Separated or divorced  Married/Civil union or living together | 12 (23.5)  8 (15.6)  31 (60.8) |
| **Mutation type** (n=24)  BRCA I or II  Lynch | 20 (83.3)  4 (16.7) |
| **Cancer type** (n=18)  Breast  Ovary  Prostate  Did not specify | 9 (50.0)  2 (11.1)  5 (27.8)  2 (11.1) |

^a^includes Asian and multiracial

**eTable 2.** User testing interview guide

| USER TESTING GUIDED INTERVIEW  There are no right or wrong answers for this part, we just want to hear your preferences and opinions. You may choose to skip questions that make you uncomfortable or stop the interview at any time. As a reminder, this interview is being recorded for transcription and research purposes. The recording will be destroyed once the study team has verified transcription accuracy.  Do you have any questions before we begin? |
| --- |
| First, we will review the CATALYST LOGIN to the WEBPAGE (Creating an Account). |
| 1. Did you have any difficulty navigating to the CATALYST webpage?   Response: ________________________________________________________________ |
| 1. Did you have any difficulty completing login?   Response: ________________________________________________________________ |
| 1. What are your thoughts about the CATALYST logo? (Color, Font, Text Size, Information Requested)   Response: ________________________________________________________________ |
| 1. What are your thoughts about the login design? How would you improve it? (Color, Font, Text Size, Information Requested)   Response: ________________________________________________________________ |
| 1. How confident are you that the CATALYST login is secure?  - Very confident - Confident - Slightly confident - Not at all confident   Response: ________________________________________________________________ |
| 1. Is it useful to have password recovery in case someone forgets their password?  - Yes - No   Response: ________________________________________________________________ |
| Now we will review the ONBOARDING (Permanent menu, Intro Video). |
| 1. Is there anything unclear about the Menu?   Response: ________________________________________________________________ |
| 1. Do you feel the Introduction Video is clear and informative? Why or why not?   Response: ________________________________________________________________ |
| Next, we will review the EDUCATIONAL VIDEOS that include an Oncologist Referral, Patient Testimonials. |
| 1. What did you like about these educational videos?   Response: ________________________________________________________________ |
| 1. What did you dislike about these educational videos?   Response: ________________________________________________________________ |
| 1. How well did the videos address your questions or concerns regarding genetic testing?   Response: ________________________________________________________________ |
| 1. What video did you like the most?   Response: ________________________________________________________________ |
| 1. What video did you dislike the most - if anything?   Response: ________________________________________________________________ |
| Now we will review the FREQUENTLY ASKED QUESTIONS (FAQ content, printable pdf of provider questions). |
| 1. Did you have any difficulty navigating the FAQ’s?   Response: ________________________________________________________________ |
| 1. Is there any information that the FAQs didn’t mention about hereditary cancer and genetic testing, that you would like to know more about?   Response: ________________________________________________________________ |
| 1. Would you use the printable pdf of provider questions? Why or why not?   Response: ________________________________________________________________ |
| 1. Do you like the format? Is it easy to follow and use?   Response: ________________________________________________________________ |
| Next, we will review the FAMILY CANCER RISK QUESTIONNAIRE (FCR). |
| 1. What are your thoughts about the language used in the Family Cancer Risk questionnaire?   Response: ________________________________________________________________ |
| 1. What are your thoughts about the formatting/design of the Family Cancer Risk questionnaire?   Response: ________________________________________________________________ |
| 1. What are your thoughts about the length of the Family Cancer Risk questionnaire?   Response: ________________________________________________________________ |
| 1. Do you think most people would fill out the Family Cancer Risk questionnaire? Why or Why not?   Response: ________________________________________________________________ |
| 1. Do you like it the way it is or have any suggestions for improving the content or design?   Response: ________________________________________________________________ |
| Next, I would like to know your thoughts about the FAMILY HISTORY QUESTIONNAIRE (FHQ, Pedigree, Risk Statement). |
| 1. What are your thoughts about the language used in the FHQ?   Response: ________________________________________________________________ |
| 1. What are your thoughts about the formatting/design of the FHQ?   Response: ________________________________________________________________ |
| 1. What are your thoughts about the length of the FHQ?   Response: ________________________________________________________________ |
| 1. If we made the FHQ optional, would you choose to complete it? Why or Why not?   Response: ________________________________________________________________ |
| 1. What are your thoughts about the design of the Pedigree?   Response: ________________________________________________________________ |
| 1. Is there anything you would add or remove from the Pedigree?   Response: ________________________________________________________________ |
| 1. Would you share the Pedigree with your provider or family? Why or Why not?   Response: ________________________________________________________________ |
| 1. Would you share the pedigree if it were shortened to only include family members with a history of cancer? Why or Why not?   Response: ________________________________________________________________ |
| 1. Is there anything unclear about the language used in the ‘Risk Statement’?   Response: ________________________________________________________________ |
| 1. How trustworthy is the information provided by Alex so far?   Response: ________________________________________________________________ |
| Now we will discuss the GENETIC TESTING READINESS and Importance Questionnaire (Readiness Ruler QNR). |
| 1. What do you think about Genetic Testing Readiness Questionnaire? What do you like or dislike about it?   Response: ________________________________________________________________ |
| 1. What are your thoughts about the language used in the question? Is there anything unclear about the language?   Response: ________________________________________________________________ |
| 1. What are your thoughts about the length of the Genetic Testing Readiness Questionnaire?   Response: ________________________________________________________________ |
| 1. What are your thoughts about the formatting/design of the Genetic Testing Readiness Questionnaire?   Response: ________________________________________________________________ |
| 1. Do you feel this Genetic Testing Readiness Questionnaire would help a person identify how ready they are to receive genetic testing and how important getting tested is to them? Why or why not?   Response: ________________________________________________________________ |
| Now we will discuss the GENETIC TESTING CHOICE section (Choice options, GT Kit QNR). |
| 1. What do you think about the way genetic testing choice options are presented?   Response: ________________________________________________________________ |
| 1. How would you feel about uploading a photo, such as an insurance card, if you wanted to order a genetic testing kit?   Response: ________________________________________________________________ |
| 1. Do you feel that Alex and the information presented up to this point, adequately informs cancer patients about hereditary cancer risk and genetic testing to help them come to a decision?   Response: ________________________________________________________________ |
| 1. How easy is this process of ordering a genetic testing kit? Are the instructions and information requested clear?   Response: ________________________________________________________________ |
| 1. What other information about hereditary cancer and genetic testing would you be looking for at this stage of your journey using Alex?   Response: ________________________________________________________________ |
| Next, we will discuss some aspects related to genetic testing RESULTS (Result Delivery, Family Communication Letter). |
| 1. Which result was delivered?    - Positive    - Variant of Uncertain Significance (VUS)    - Negative |
| 1. How do you feel about the positive result delivery method? Is there anything unclear?   Response: ________________________________________________________________ |
| 70b. How do you feel about the VUS result informational video? Is there anything that is not clear?  Response: ________________________________________________________________ |
| 70c. How do you feel about the negative result information provided? Is there anything unclear about it?  Response: ________________________________________________________________ |
| 1. How do you feel about the [Positive/VUS/Negative] Family Communication Letter?   Response: ________________________________________________________________ |
| 1. Do you feel the Family Communication Letter would help facilitate a conversation about hereditary cancer and your genetic test results (that is if you got tested) between you and your family? Why or Why not?   Response: ________________________________________________________________ |
| I will now ask about your USER PREFERENCES (Notifications/Reminders, Voice Chat). |
| 1. What are your thoughts about the language/content/frequency of the Email/Text notification/reminders?   Response: ________________________________________________________________ |
| 1. Do you think Voice Chat is a useful tool for a user?   Response: ________________________________________________________________ |
| 1. What did you like better, using the Voice Chat feature or text? Why?   Response: ________________________________________________________________ |
| 1. What do you think about the tone of Alex’s voice? Would you change it? If so, how and why?   Response: ________________________________________________________________ |
| 1. What other User Preferences would you suggest for us to include on this platform?   Response: ________________________________________________________________ |
| Next, we will discuss the TABS (Chat vs. Questionnaire) |
| 1. What do you think about the design of the Chat tab? (Color, fonts, text size)   Response: ________________________________________________________________ |
| 1. Is there anything you would add or remove from the Chat tab?   Response: ________________________________________________________________ |
| 1. What do you think about the design of the Questionnaire tab?   Response: ________________________________________________________________ |
| 1. Is there anything you would add or remove from the Questionnaire tab?   Response: ________________________________________________________________ |
| Next, we will discuss Alex, the Genetic Cancer Risk Evaluation (CARE) Digital Health Guide (Design, Persona, Tone, Personality, Language) |
| 1. What are your thoughts about Alex’s design? (Colors, fonts, text size)   Response: ________________________________________________________________ |
| 1. What are your thoughts about interacting with Alex (persona)?   Response: ________________________________________________________________ |
|  |
| Please indicate how much you agree or disagree with the following statements. |
| 1. I think this digital health guide, Alex, could help cancer patients who may have hereditary cancer decide whether or not to get genetic testing.  - Strongly Agree - Agree - Disagree - Strongly Disagree |
| 1. I would recommend this digital health guide, Alex, to other cancer patients who are at increased risk for hereditary cancer.  - Strongly Agree - Agree - Disagree - Strongly Disagree |

**eTable 3. Error occurrence and researcher intervention during prototype usability testing**

| **Functionality (Chatbot Flow)** | **Error Occurrence** | **Researcher Intervention** |
| --- | --- | --- |
| Log-in (Activation Email, Password) |  | X X X X |
| Account Creation | X |  |
| Onboarding (Permanent Menu, Introductory Video) | X X | X |
| Educational Videos (Oncologist Referral, Patient Testimonial) | X |  |
| Frequently Asked Questions (FAQs, Questions for Provider) |  |  |
| Family Cancer Risk Questionnaire (FCQ) |  | X |
| Family History Questionnaire (FHQ) |  | X |
| Pedigree/Family Tree | X X X X | X |
| Readiness Ruler |  |  |
| Genetic Testing Choice (Choice Options, GT Kit QNR) |  | X X X X |
| Results Delivery |  |  |
| Family Communication Letter |  |  |
| User Preferences (Notifications/Reminders) | X |  |
| Voice Chat | X X |  |
| Tabs (Chat/Questionnaire) | X X X X X X |  |
| DHG Knowledge Base |  |  |

X reflects the occurrence of an error/researcher intervention.
